# Adaptable and Automated Small UAV Deployments via Virtualization

**DOI:** 10.3390/s18124116

**Published:** 2018-11-23

**Authors:** Borja Nogales, Victor Sanchez-Aguero, Ivan Vidal, Francisco Valera

**Affiliations:** 1 Telematic Engineering Department, Universidad Carlos III de Madrid, Avda. Universidad, 30, 28911 Leganés (Madrid), Spain; victor.sanchez@imdea.org (V.S.-A.); ividal@it.uc3m.es (I.V.); fvalera@it.uc3m.es (F.V.); 2 IMDEA Networks Institute, Avda. del Mar Mediterráneo, 22. 28918 Madrid, Spain

**Keywords:** Small Unmanned Aerial Vehicles (SUAVs), Network Functions Virtualization (NFV), Adaptable SUAV deployments

## Abstract

In this paper, we present a practical solution to support the adaptable and automated deployment of applications of Small Unmanned Aerial Vehicles (SUAVs). Our solution is based on virtualization technologies, and considers SUAVs as programmable network platforms capable of executing virtual functions and services, which may be dynamically selected according to the requirements specified by the operator of the aerial vehicles. This way, SUAVs can be flexibly and rapidly adapted to different missions with heterogeneous objectives. The design of our solution is based on Network Function Virtualization (NFV) technologies, developed under the umbrella of the fifth generation of mobile networks (5G), as well as on existing Internet protocol standards, including flying ad hoc network routing protocols. We implemented a functional prototype of our solution using well-known open source technologies, and we demonstrated its practical feasibility with the execution of an IP telephony service. This service was implemented as a set of virtualized network functions, which were automatically deployed and interconnected over an infrastructure of SUAVs, being the telephony service tested with real voice-over-IP terminals.

## 1. Introduction

The progressive evolution witnessed by the small unmanned aircraft systems during the last ten years, in conjunction with the services and products that can be offered by this kind of systems, have led to their consideration as an earnest candidate to support the development of new emergent civilian applications. A small unmanned aircraft system includes a Ground Control Station (GCS) that monitors and controls the operation of a set of Small Unmanned Aerial Vehicles (small UAVs or SUAVs). These SUAVs may have different capabilities (e.g., through an heterogeneous set of on-boarded sensors), and can be deployed over a delimited geographical area to provide a particular service. In contrast to a usual use case where the services provided by SUAVs are oriented towards the collection of data (e.g., sensor values, video images, etc.), as well as their transmission to a ground station, the research community and the industry have recently considered the development of new value-added services and applications where SUAVs play a fundamental role. Among these, we can mention remote sensing in surveillance operations [1], agribusiness [2], collaborative search and rescue [3,4,5,6,7], building aerial sensor networks to aid disaster management [8], or supporting backbone communications to mobile ground stations [9].

On the other hand, the advent of the new generation of mobile networks (i.e., 5G) imposes new and stringent requisites to guarantee a fair balance between speed, latency and cost of communications, which are expected to be potentially originated by millions of connected devices. For that purpose, the shared use of resources, allowing the dynamic provision of network functions, is foreseen as one of the key enablers of this new generation of networks. In this context, the Network Functions Virtualization (NFV) paradigm presents an innovative solution in the sector of information and communication technologies, aiming at supporting the active and high-performance processing of traffic delivered across 5G networks.

**Figure 1:**
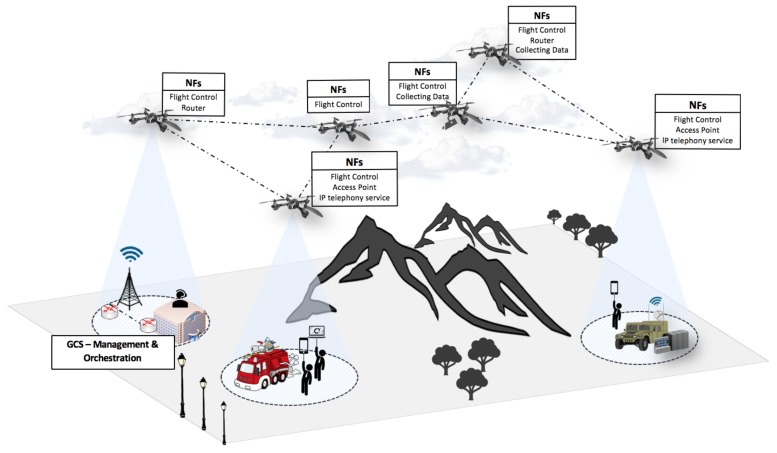
Deployment of SUAVs offering diverse network functionalities (NFs).

Under the above considerations, this paper presents a practical approach based on NFV technologies to support SUAV deployments that can be rapidly adapted to civilian missions with different objectives. In this approach, SUAVs offer an NFV programmable infrastructure that enables the agile integration of services and functions, which may be determined by the operator of the SUAVs at deployment time. As an example (as shown in Figure 1), SUAVs could be deployed to provide a communications infrastructure over a disaster area, where networking facilities are insufficient or unavailable (e.g., due to an earthquake or during firefighting activity). In this example, a set of SUAVs could be used to deploy virtualized access points and routing functions, creating an aerial communication infrastructure over the deployment area. In addition, some SUAV units could execute the diverse components of an IP telephony service, thus enabling real-time voice and video communications among the ground units operated by the emergency response team. Some aerial vehicles could be configured to obtain and disseminate video images and temperature readings, improving the situational awareness and, consequently, the effectiveness of the adopted response actions. Furthermore, the same SUAVs could later be used in a different mission, such as a search and rescue operation in a remote area. In this case, the flight control unit carried by the SUAVs could be upgraded for certain aircrafts in the deployment, with the purpose of tracing different flight trajectories for an efficient inspection of a delimited search area.

Nonetheless, we want to highlight that the full realization of this vision is challenging due, among other reasons, to the limited capacity of the hardware and software platforms that can typically be on-boarded on SUAVs; the need to automatically manage the resources provided by that platforms and deploy virtual functions on top of them, despite being transported by flying vehicles; the requirement to specify appropriate placement policies for virtual functions (e.g., to indicate which virtual functions should be executed over the same SUAV unit); and the energy consumption of the battery-powered SUAVs, which is a limiting factor of the operation time.

This article continues a research line initiated in [10], where we presented the preliminary design of an NFV system capable of deploying network services over SUAV platforms, and we demonstrated the practical feasibility of this design. The work presented in this article extends our previous research covering the following aspects:

- It extends the original design of the system, incorporating specific mechanisms to support wireless control communications between the GCS and the NFV infrastructure provided by the SUAVs.
- It provides a new design for the SUAV device, which does not require specific routing functions at the SUAV to handle the traffic exchanged by the virtual functions hosted by the device.
- It provides a new mechanism to support data-plane communications. This mechanism is based on virtual networks that are established over the aerial network infrastructure provided by the SUAVs, which now run a flying ad-hoc network routing protocol such as Ad hoc On-Demand Distance Vector (AODV) [11] or Optimized Link State Routing (OLSR) [12]. This simplifies the definition of the services that are to be virtualized and eases the reconfiguration of the aerial network formed by the SUAVs.
- It presents a functional prototype of the platform with the defined extensions, as well as a validation of the solution with the deployment of a realistic network service (an IP telephony service).
- Finally, it evaluates the performance that may be offered by different flying ad-hoc network routing protocols to support control and data communications through the network formed by the SUAV devices.

The rest of this paper is organized as follows. After reviewing the existing literature on the utilization of SUAVs to support network functionalities and softwarization in Section 2, the main challenges related with the utilization of NFV technologies in SUAV environments and the design of our proposed NFV system are detailed in Section 3. Section 4 presents our prototype implementation and the tests that have been done to validate our system design. Finally, Section 5 concludes the paper and presents the short-term directions of our work.

## 2. Related Work

The softwarization of network functions, or NFV, is currently considered a key component in the context of the fifth generation of mobile networks (5G networks). In this paper, we consider this technology as an enabler with the potential to support the flexible and adaptable deployment of functions and services over a set of SUAVs. In this context, the European Telecommunications Standards Institute (ETSI) is leading the standardization activities on NFV, which encompasses the definition of a reference architectural framework [13] for NFV deployments.

Among the main reference blocks of this architecture, the Virtual Network Function (VNF) is the element in charge of providing the functionality of a network component (e.g., a residential gateway, an access point, a firewall, etc.) through a software implementation. The description of a VNF in terms of its deployment and operational behaviour is covered by the Virtual Network Descriptor (VNFD). A set of interconnected VNFs creates a Network Service (NS), e.g., a number of virtual network routers may provide a data communication service to end users. In this case, the Network Service Descriptor (NSD) defines the characteristics of an NS, including its constituent VNFs and the interconnections between them. Secondly, the NFV Infrastructure (NFVI) provides the hardware and software resources that are needed to deploy, execute and manage the VNFs. These include hardware resources such as computing, storage and networking, which are allocated to the VNFs through a virtualization layer abstraction that enables the decoupling of the VNF software from the underlying hardware. Finally, the Management and Orchestration (MANO) framework supports the proper coordination of all the operations carried out in the NFV environment. These operations enable the automated deployment of network services composed by the VNFs over the NFVI. For this purpose, the definition of the MANO framework contains three functional elements: (1) the Virtual Infrastructure Manager (VIM), controlling the hardware and software resources of the NFVI; (2) the VNF Manager (VNFM), which handles the life cycle of VNFs; and (3) the NFV Orchestrator (NFVO), in charge of building up end-to-end services, and coordinating with the VNFM and VIM functions the available resources and the deployment and configuration of the VNFs.

On the other hand, the utilization of softwarization and cloud technologies has already been considered in resource-constrained environments, such as those contemplated in the context of the Internet of Things (IoT). In this respect, different works in the literature focus on the definition of an IoT cloud model to enhance the scalability of traditional wireless sensors networks. The work in [14] presents a mathematical model to virtualize the physical resources offered by a sensor node. This work proposex a paradigm shift in applications based on wireless sensor networks, to consider cloud platform technologies. In [15], the authors described an interactive location-based model for IoT, which uses the cloud to enable the collection of sensor data on demand according to the locations of interest established by the user. The work in [16] introduces an interactive model to support an IoT cloud capable of offering sensing services on demand at the same time, but with different latency requirements.

Focusing on the provision of network functionalities and softwarization in UAV environments, even though these aspects have received consideration by the research community, our review of the recent literature reveals that no solution has been proposed to date to support the agile integration of services and functions into UAVs, or to deploy adaptive communications infrastructures based on these devices. In [17], the authors presented a recent and detailed review of the state of the art on UAV-based communication networks. In [18], the authors proposed the use of a cloud application for monitoring UAVs and adjust flight trajectories to avoid collisions. The work in [19] contemplatex UAVs as devices belonging to a computing platform, capable of providing services through communications with other UAVs or with ground units. These services were provisioned using middleware technologies. However, the authors did not consider the limitations in computing capacity that these devices typically present or the use of virtualization or NFV. In [20], the authors described the utilization of UAVs as *OpenFlow* switches, although they did not consider the deployment of functions over the UAVs themselves. In [21], the use of a UAV is presented as a communications relay to establish communications between a GCS and other UAVs. This work considers UAVs as units with limited computing capacity by incorporating single board computers (SBCs), which are used to support the information relay and the mission control functionalities. In [22], the paradigm of function softwarization in an irrigation system based on a network of sensors and UAVs is considered. However, the UAVs are not used as computing nodes to execute functions. In [23], VNFs are placed in the GCS to execute *routing* functions, although their deployment is not automated as the work does not contemplate an orchestration platform.

## 3. System Design

As previously mentioned, our work studies the applicability of virtualization technologies and standards to build flexible SUAV deployments, capable of agilely adapting to different missions in the civilian scope. With this objective, we consider the utilization of NFV, along with general purpose hardware and software platforms that can be on-boarded into small aerial vehicles. These platforms will provide the underlying substrate to execute network, transport and application level functions, which will be implemented in software and deployed over the SUAVs as required using virtualization technologies.

The use of NFV technologies in SUAV environments offers a number of potential advantages: (1) the ability to flexibly adapt the functions offered by SUAVs to the specific mission requirements or objectives; (2) the capacity to agilely deploy moderately complex SUAV services and applications, installing and configuring the necessary components within a limited time period; (3) it facilitates the development of new functions and services, with the possibility of testing them in controlled environments with similar characteristics compared to their corresponding production environments, as well as evolving and replacing them quickly; (4) the provision of open platforms, which offers new possibilities for the incorporation of developers and manufacturers, including other communities in the sector of information technologies and communications to promote innovation; (5) the flexibility to accommodate deployment scenarios with changing resource demands, for example by incorporating new virtualized functions or scaling them as it becomes necessary; and (6) in general, reduction of investment and maintenance costs, through the use of general purpose platforms (e.g., open platforms), whose manufacturing and supply costs can be reduced by economies of scale.

Despite the aforementioned potential benefits of using NFV in the context of SUAVs, we want to note that it also introduces a series of technical challenges that must be carefully considered. On the one hand, the hardware equipment that can be on-boarded in an SUAV, providing the underlying substrate to support the execution of virtualized functions, is typically limited in terms of size and weight. This is especially evident in the case of the small-sized UAVs that have recently emerged in the market, which could at best carry a small set of Single-Board Computers (SBCs). In this way, the hardware platform for the execution of virtual functions can present significant limitations in terms of computing capacity, networking and storage. On the other hand, such hardware must be integrated as part of the NFV infrastructure of an existing VIM solution, in such a way that it can be used by an NFV orchestration service to deploy virtualized functions. Additionally, it should be possible to make an appropriate distribution of VNFs to SUAVs, in order to indicate which VNFs should be executed on the same aircraft. Moreover, the control communications that are necessary to manage the infrastructure resources carried by the SUAVs should be maintained, even when these devices are flying. Finally, we must consider that the UAVs have a battery, and therefore a limited operating time. For this reason, specific mechanisms may be required to support the replacement of these devices, including the migration of the virtualized functions hosted by them to new or existing SUAV units. We must bear in mind that these requirements are not common in traditional virtualization platforms, where compute nodes are typically high performance servers, installed in a data-center, and interconnected through a high capacity fixed network technology (e.g., an ethernet network).

**Figure 2:**
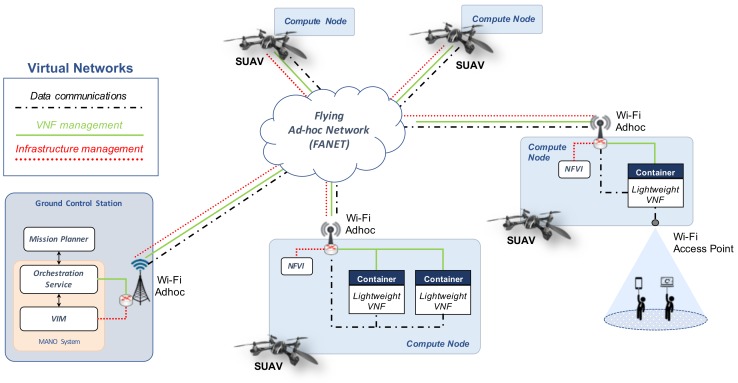
Overview of the platform design.

Given the complexity of these challenges and to guarantee the architectural and technological convergence of our work with existing solutions, our proposal has been designed in accordance with recognized standards in the NFV arena. In particular, we consider the NFV reference architecture proposed by ETSI [13], which is reviewed in Section 2. Figure 2 illustrates a conceptual vision of our solution, serving as the basis for the definition of its corresponding architectural design. Taking into account the capacity limitations of the typical hardware platforms that can be on-boarded on SUAVs, the Virtualized Network Functions (VNFs) should be developed to provide their intended functionality, but making a responsible use of the available resources. For this reason, in the vision illustrated in the figure, these VNFs are referred to as lightweight VNFs. In general terms, the design of our solution is structured into three main components.

1. The MANO system, located in the GCS, is the component in charge of the management and orchestration of available hardware and software resources, as well as the deployment and interconnection of the lightweight VNFs. It provides an orchestration service, with the execution of an NFV Orchestrator (NFVO) and a VNF Manager (VNFM), and a Virtual Infrastructure Manager (VIM). The GCS offers a stable environment with appropriate resources for the operation of this component, which is convenient given its criticality.
2. The hardware and software infrastructure that supports the execution of lightweight VNFs, i.e., the NFVI. As we have already mentioned, this infrastructure will be on-boarded in the SUAVs.
3. The mission planner, also located at the GCS, which is the entity responsible for specifying the descriptors of the network services to be deployed, each one as a composition of VNFs, in addition to the configuration parameters of each VNF and the policies that determine the allocation of the VNFs to the SUAVs. The mission planner generates this information in concordance with the mission requirements that are provided by the operator of the SUAVs at deployment time.

Focusing on the architectural design of the SUAV device, each SUAV carries a general purpose hardware and software platform. This platform, hereafter referred to as a compute node following cloud computing terminology, has a wireless communication interface that enables the exchange of data with every other component of our design that is within the radio coverage of the compute node (i.e., every other compute node and the MANO system). This wireless interface can be based on different technologies, e.g., it may provide a line-of-sight or Wi-Fi radio link. Additionally, as reflected in the figure, some compute nodes may include a secondary network interface to provide wireless access connectivity to ground units.

In our design, the compute nodes on-boarded at the different SUAVs form a wireless ad-hoc network that enables multi-hop data communications (that is, communications among compute nodes at different SUAV units, and between the compute nodes and the MANO system). These data communications across the ad-hoc network are supported with the execution of a Flying Ad-hoc Network (FANET) routing protocol at each compute node (e.g., AODV [11] or OLSR [12]) (we assume that the support of this routing protocol is enabled at each SUAV as a prior step to an operational deployment). Besides, to isolate the different traffic types exchanged over this wireless ad-hoc network, our solution defines a set of virtual networks that operate on top of the wireless ad-hoc network.

In particular, to support management operations towards the compute nodes (e.g., to monitor the available resources at each compute node, to instantiate and configure a VNF instance, or to terminate that instance), each compute node included in the NFV infrastructure supports two types of communications: (1) communications between the VIM and the compute nodes (labeled as *Infrastructure management* in Figure 2), to control the computing, storage and networking resources of the compute nodes; and (2) communications between the Orchestration Service and the VNFs (denoted as *VNF management* in Figure 2), to manage the configuration and the lifecycle of the lightweight VNFs. In our design, each of these types of management communications is delivered over an independent virtual network, which is created over the wireless ad-hoc network infrastructure offered by the SUAVs (i.e., the virtual network operates over the FANET routing protocol executed at every SUAV). Thus, management communications are isolated and delivered “in-band”, through the aerial network infrastructure conformed by the compute nodes. Finally, given that management communications are always necessary for any application requiring the deployment of an unmanned aircraft system, both virtual networks can be pre-configured offline at each SUAV unit and the GCS.

Regarding data communications among the lightweight VNFs that conform a network service (indicated as *Data communications* in Figure 2), these are also isolated using virtual networks that are built on top of the wireless ad-hoc network established by the compute nodes. The number and configuration of these virtual networks will typically be application-specific (e.g., IP telephony traffic would require an independent virtual network). Hence, they will automatically be created by the MANO system as required, under the indication of the Mission Planner.

In an operational deployment, the VNFs of the network service (e.g., the components of an IP telephony service) would be instantiated and configured at every involved compute node. In addition, the MANO system would also create the virtual networks that are necessary to interconnect the VNFs and support data communications. In our solution, the flight control functionalities required at each SUAV unit can be provided by a lightweight VNF. This VNF will execute the needed actions to position the SUAV at its target position (the position, as well as the trajectory of the SUAV to reach that position, can be configured at the flight control VNF by the MANO system). Management communications (i.e., between the MANO system and each VNF, and between the VIM and each compute node) are still maintained through the virtual networks that have been pre-configured offline for this purpose, with the support provided by the FANET routing protocol that operates at the compute nodes.

Finally, as a specific design criterion regarding the deployment of virtual functions, we want to highlight that, given the limited-capacity hardware and software platforms that can be provided and/or transported by SUAVs, we have decided to use container virtualization, as opposed to traditional hypervisor-based virtual machines, to support the deployment of the lightweight VNFs in our design. This is a crucial consideration that has a significant impact on the practical feasibility of the proposed design.

## 4. Validation of the Solution

In this section, we describe the most relevant aspects regarding the validation of our solution. Starting from the basis of our previous work, we have evolved the preliminary implementation presented in [10] to provide a functional prototype of the design presented in Section 3. In addition, we have developed a number of lightweight VNFs, to support the creation of moderately complex network services that have served to test and validate the proposed design. The system prototype and the lightweight VNFs have been implemented using open-source software technologies. In addition, we have evaluated the feasibility of using different FANET routing protocols through simulation, to support the control and data communications offered by the virtual networks of our design.

### 4.1. Prototype Implementation

The use of open-source technologies was considered one of the basic principles that used throughout the implementation process. In particular, we selected Open Source MANO (OSM) Release FOUR [24] to provide the functionalities corresponding to the orchestration and the lifecycle management of lightweight VNFs. With respect to the VIM, we utilized the set of software tools provided by the well-known cloud computing platform OpenStack Ocata [25]. Both the OSM stack and the VIM provide the MANO system of our design, and were deployed over a virtual machine running in a mini-ITX computer (Intel Core i7 2.3 GHz, 16 GB RAM, 128 GB SSD, 4 GbE ports).

Regarding the SUAV platforms, we used a number of aerial vehicles DJI Phantom 3 [26], each carrying a single board computer Raspberry Pi 3 Model B (RPi). These RPis are used as the compute nodes of our design, supplying the needed resources in terms of computing, storage and networking, and supporting the execution of the lightweight VNFs. We incorporated the RPi boards as compute nodes of the OpenStack VIM, doing the necessary configurations to enable virtual networking through Linux networking bridges. Besides, each RPi includes an integrated Wi-Fi interface, and a number of them also contain a secondary wireless interface provided by a Wi-Fi USB adapter. The first interface enables air-to-air and air-to-ground ad-hoc communications with other SUAVs and with the MANO system, respectively. The second interface is intended for deploying a wireless access point, capable of providing network access connectivity to mobile ground units (in a prior research work [27], we validated the suitability of these multi-interface devices to support multimedia communications).

With respect to the virtual networks that enable both control and data plane communications that take place in our platform, we used Virtual eXtensible Local Area Networks (VXLAN) [28]. VXLANs present a feasible solution to exchange control and data traffic over a wireless ad-hoc network since: (1) the VXLAN traffic can be sent over the Wi-Fi interface in ad-hoc mode that is available at every compute node (directly sending traffic from a lightweight VNF, deployed over a virtualization container, through a Wi-Fi ad-hoc network is challenging, as this is not currently supported by the Linux kernel of the RPis); (2) they can be dynamically created by the VIM, as instructed by the OSM stack, to interconnect lightweight VNFs hosted by different compute nodes; and (3) the utilization of VXLANs does not require additional network configurations (e.g., network routes) at the intermediate RPi boards that conform the network path between two communicating entities (e.g., between two VNFs).

**Figure 3:**
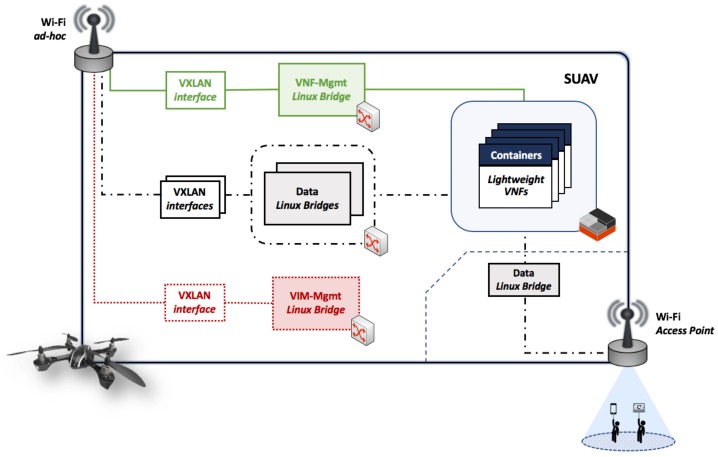
Compute node network configuration.

Figure 3 details the network configuration that has been done at each compute node. Firstly, a VXLAN interface attached to a Linux bridge (VIM-Mgmt in the figure) enables the control communications required by the VIM to manage the computing, storage and networking resources available at every compute node. Secondly, an additional VXLAN interface allows the MANO system to carry out the lifecycle management of the lightweight VNFs hosted by the SUAVs. In this case, the Linux bridge attached to the VXLAN interface (VNF-Mgmt in the figure) allows communications with every lightweight VNFs running at the same compute node. Note that both VXLANs are statically pre-configured at every compute node to enable the control communications of our system, as presented in Section 3. Finally, the configuration of the compute node allows the dynamic creation of virtual networks for data communications among VNFs (residing at the same, or at different compute nodes), as requested by the VIM and according to the instructions provided by the OSM stack.

### 4.2. Validation Scenario

**Figure 4:**
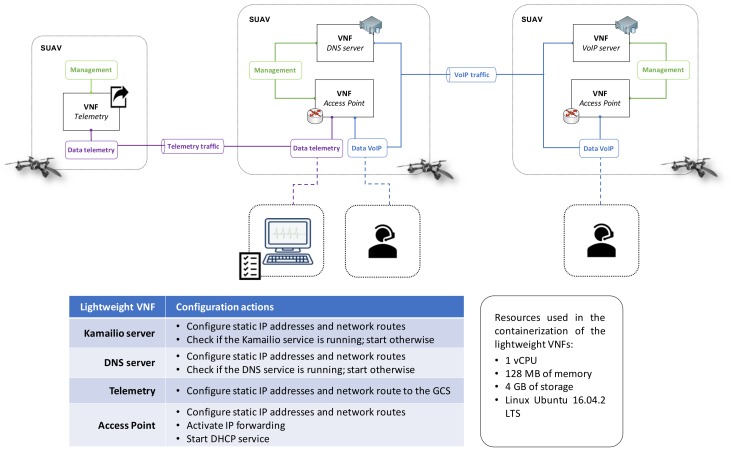
Validation scenario.

This section describes the experiment carried out to validate the potential of our platform to execute realistic and moderately complex network services. In this experiment, we implemented the Network Service (NS) shown in Figure 4, including the NFV descriptors (NSD and VNFDs) and the set of lightweight VNFs that conform the NS. The NS provides the functionality of an IP telephony service, and supports the delivery of telemetry from every SUAV to an equipment in the ground control station of the SUAVs. The NFV descriptors and the lightweight VNFs have been developed and made available under an open source license (https://github.com/5GinFIRE/mano/tree/master/descriptor-packages).

Regarding the lightweight VNFs that enable the provision of the IP telephony service, the NS includes two virtual access points (Access Point VNFs in Figure 4), which support data communications between end user equipment (i.e., ground units) located at different areas. Each of these virtual access points holds a Dynamic Host Configuration Protocol [29] (DHCP) server, which automatically provides the required network configuration to allow IP access connectivity to connected users. On the other hand, the IP telephony service enables the exchange of signalling traffic to establish multimedia sessions between communicating endpoints, i.e., Voice over IP (VoIP) calls among user equipment at ground units. To support this service, we developed a lightweight VNF providing the functionality of a VoIP server, based on the open source software Kamailio [30]. This VoIP server VNF enables the establishment of voice communications with the utilization of the Session Initiation Protocol (SIP) [31]. Finally, a lightweight VNF provides the functionality of a Domain Name System [32] (DNS) server, resolving host names to IP addresses during the execution of the IP telephony service.

On the other side, the exchange of telemetry information (GPS positioning, information from on-boarded sensors, etc.) from an SUAV to a ground station is allowed by the lightweight VNF referred to as *Telemetry* in Figure 4. In this case, we emulated the transmission of telemetry data using the Iperf tool [33], which enables the generation of different data streams from a source to a destination. In our test scenario, telemetry information was emulated as a User Datagram Protocol (UDP) [34] stream with a bandwidth of 32 kbps.

With this, we instantiated the NS over our SUAV platform using the client application of OSM. This instantiation results in the execution of the aforementioned VNFs in virtual containers at the SUAVs, following the disposition indicated by Figure 4. For that end, the definition of each SUAV as an availability zone (i.e., a set of resources) in the OpenStack VIM allowed us to execute each VNF at the expected SUAV through the placement policies provided by OSM. The lightweight VNFs composing the IP telephony service communicate through a VXLAN that is established over the Wi-Fi ad-hoc network. Telemetry information is exchanged with the GCS using a different VXLAN (over the Wi-Fi adhoc network as well). Both VXLANs are dynamically created by the VIM, as instructed by the OSM stack. This way, and according to our system design, telemetry traffic is isolated from the VoIP traffic. Besides, the configuration of lightweight VNFs after their deployment (commonly known as Day-1 configuration) is done through Ansible playbooks [35], using a specific open-source development contributed by the authors of this paper to the OSM community (https://osm.etsi.org/wikipub/index.php/Example_VNF_Charms). The configuration operations for each lightweight VNF, as well as the virtual resources required for their execution, are summarized in Figure 4.

Once the deployment and the configuration of the lightweight VNFs was completed, we connected a wireless VoIP phone ZyXEL Prestige 2000W to each of the access points offered by our NS, with the objective of carrying out a VoIP call. In addition, a commodity laptop was connected to the access point involved in the operations related with the exchange of telemetry information, emulating an equipment at the GCS.

**Figure 5:**
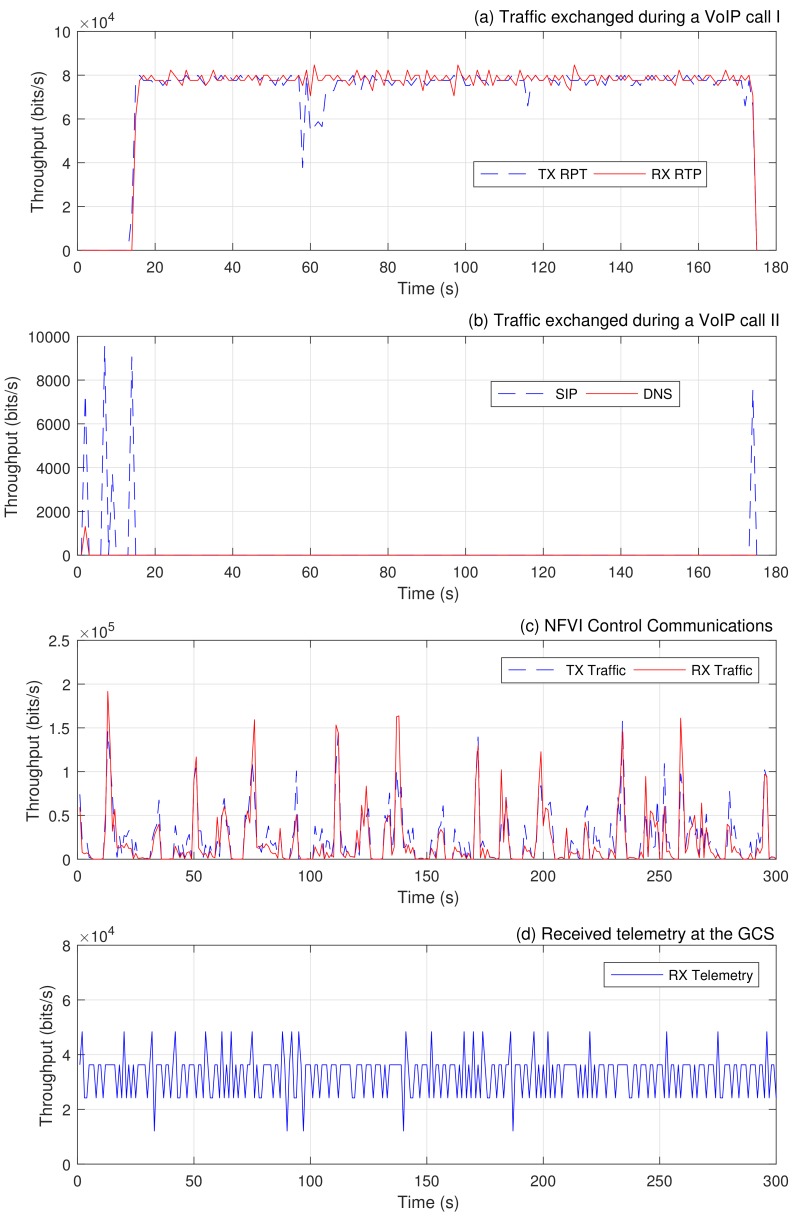
Validation measurements.

Figure 5a,b illustrates the traffic exchanged during the VoIP call established by the VoIP terminals, including the DNS and the SIP signalling messages needed to execute the call. The figure also reflects the voice traffic transmitted and received by one of the wireless phones. The call was made without errors and with appropriate sound quality. On the other hand, Figure 5c represents the control traffic generated and received periodically by the MANO system, which is exchanged in order to monitor and synchronize the information of the available/consumed resources at each compute node. This traffic was measured to get an insight on how control traffic operates in our platform once an NS is being executed, and to verify how this traffic may affect the provided service. As expected, the measurements show that this traffic is negligible (with an average of 20.65 kbps) compared with the traffic exchanged during the execution of an NS, e.g., during the VoIP call. Finally, Figure 5d shows the bandwidth consumption of a telemetry stream received at the ground control station (i.e., at the laptop), originated by the Telemetry VNF of one of the SUAVs.

### 4.3. Ad-Hoc network Routing Alternatives

As we have mentioned, control communications in our system are enabled through virtual networks that may eventually work on top of a FANET routing protocol that allows the creation of distributed networks where device to device (D2D) communications take place. As presented in [17], there are different alternatives to deploy these FANETs either based on extensions to the current algorithms and protocols designed for Mobile Ad-hoc Networks (MANET) and Vehicular Ad-hoc Networks (VANET) (see also [36,37,38,39]) or based on more innovative solutions based on Software-Defined Network (SDN) and NFV technologies to provide a connectivity backbone (see [23,40,41] for instance). However, it is still unclear that those solutions are valid for the special features that SUAV networks present (in particular their fast mobility capabilities that implies quick topological changes). The aim of this subsection is to validate, through a simulation environment, the usability of this type of protocols for the scenario that is proposed in this paper. We consider for this purpose two of the most popular FANET standard protocols:

1. The standard Ad-hoc On-Demand Distance Vector (AODV) protocol [11] is one of the most popular Reactive Routing Protocols (RRP). In AODV, routes are determined whenever a source node needs to communicate with a destination node. Each node only maintains the routes that are in use. In scenarios with changing topology, finding a new path may take a long time, eventually leading to a delay increase or packet loss.
2. The standard Optimized Link State Routing Protocol (OLSR) [12] is one of the most popular Proactive Routing Protocols (PRP). In OLSR, flying nodes store the updated list of destinations and their routes. OLSR has mechanisms to periodically refresh routing tables to maintain the latest network topology information. The main problem resides in the consumed resources to maintain an updated routing table and even more on possible unused routes. In limited bandwidth scenarios, this type of protocols may present some restrictions.

#### 4.3.1. Scenario Definition

**Figure 6:**
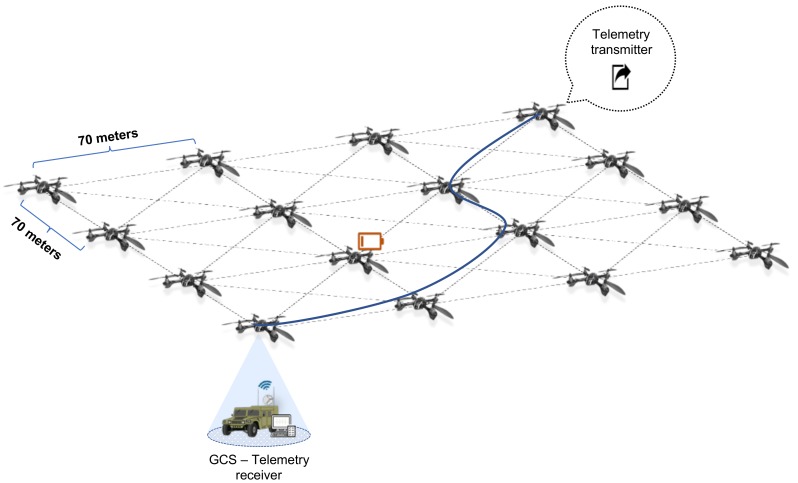
FANET for simulation scenario.

**Figure 7:**
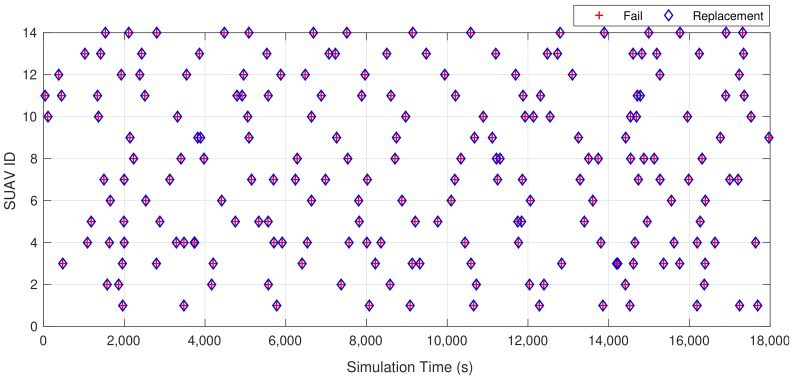
Example of fails and replacements of one simulation.

To validate the usage of both protocols, we considered the simulation scenario depicted in Figure 6, highlighting that the included SUAV cloud platform represents a FANET. In this scenario, 16 SUAVs placed in a grid with a constant position with a separation of 70 m (maximum distance reached in the simulation implementation with a reasonable link quality which covers an area bigger than 78,400 m^2^) compose the FANET with the aim of providing a network service during 1 h (3600 s). With respect to the SUAVs batteries, we assumed that those are mainly limited by flight energy consumption since other energy components demanded by the network services (e.g., AP, DCHP or Routing functionalities being executed by the SUAVs) are not comparable with the energy required by flight operations. For this simulation, we established a service time of around 20 min (1200 s time between failures) for each SUAV (battery lifetime) based on the technical specifications of the DJI Phantom 3 model [26]. In a second test, we reduced the time between failures to 5 min to get insight into the behavior of the selected protocols in extreme (unrealistic) conditions. Since battery life can be affected by different factors such as environmental conditions, maximum charge or flight conditions, we used a uniform distribution to model it characterized by the range [a=0,b=40], which provides an unfavorable dispersion of values with an average of 20 min. In a typical mission, when an SUAV battery is under a certain threshold, the SUAV will fly back to the GCS to replace it and another SUAV will come onto the scene to provide the service of the replaced SUAV. Normally, this replacement time is quite low since battery failures can be predicted from the GCS and the new SUAV can be sent to perform the replacement in advance. For that reason, we used an exponential distribution λ=1/3, which gives an average replacement time of 3 s to model these low replacement times. Figure 7 shows an example of fail and replacement events for one of the performed simulations (note that the SUAV acting as AP and the one transmitting the telemetry information are not failing during the simulation since they are assumed to be statically positioned and the only power consumption is due to the network function provisioning).

To perform the simulations on the wireless ad-hoc networks with different mobile nodes, we used Network Simulator 3 [42] (ns-3 provides a discrete-event network simulator for Internet systems). Specific existing modules for ns-3 are used to simulate the FANET protocols. Node movements have been calculated in advance in order to approximate them as much as possible to a real SUAV application. These individual movement traces are later installed on each node to perform the simulation. The network interfaces available in the different SUAVs are common IEEE 802.11b interfaces.

To measure the network performance, we set an Iperf flow between two edge nodes (UDP flow with a rate of 32 Kbps) emulating the telemetry information sent by one of the SUAV (denoted in Figure 6 as telemetry transmitter) that we have previously mentioned during the experimental validation in Section 4.2. All the simulation details can be found in Table 1.

#### 4.3.2. Simulation Results

**Figure 8:**
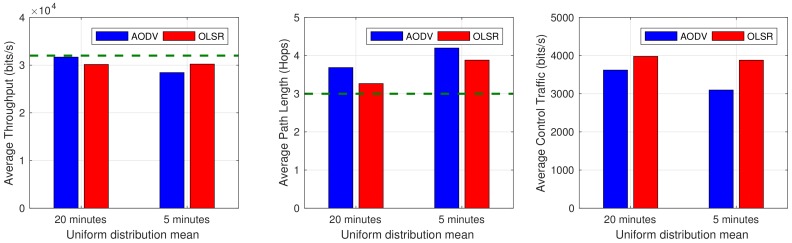
Simulation results.

To compare the different protocols, we defined three relevant metrics: the convergence time needed to find a path between two nodes (characterized in fact by the average total throughput, meaning that the less throughput achieved, the longer it takes to converge), the overhead that is generated by the routing protocol (overall total control traffic) and the optimal path usage measured in term of the percentage of packets that are sent through the shortest path. All the configurable parameters and timers for AODV and OLSR are based on the default values indicated, respectively, by Perkins et al. [11] and Clausen and Jacquet [12]. After ten simulations (results are the average of all the experiments), we can conclude that both protocols may be a suitable alternative for this scenario. Convergence times, represented by the throughput, are quite similar (slightly better in AODV with failures every 20 min, although OLSR performs better when failures are increased, as shown in Figure 8a). OLSR normally selects shorter paths, as illustrated in Figure 8b, both for the realistic scenario and the worst case scenario. In fact, this is quite reasonablem since OLSR, as RRP, is converging to the optimal path when it is available, as opposed to AODV that maintain the selected path until a failure takes place. As expected, the overhead introduced by AODV (Figure 8c) is smaller than the overhead introduced by OLSR, however, both are negligible compared with the regular service throughput.

## 5. Conclusions

In this paper, we present a solution to support the flexible, automated and cost-effective deployment of network services over small unmanned aerial vehicles. This enables providing diverse and heterogeneous applications of SUAVs over delimited geographic areas, which can be flexibly specified by the operator of the SUAVs prior to the deployment. Our solution continues a previous research on this field [10], providing an improved design and a functional prototype of the system, which has been validated through the implementation, deployment and execution of an IP telephony and a Telemetry service over a set of SUAVs. In addition, we also validated the usage of different FANET routing protocols in our scenario to enable any-to-any communications over the aerial network formed by the SUAVs. As a conclusion of this study, both alternatives, AODV and OLSR, are able to fulfill the requirements to be selected as suitable routing protocols for future implementations.

In the short-term, our work aims at developing new mechanisms to support the seamless replacement of SUAV units, e.g., due to battery constraints, with the migration of the virtualized network functions hosted by those SUAVs to other aerial vehicles. Migration of container-based VNFs requires a careful consideration, as containers are intended for stateless microservice-style applications, which inherently hinders the migration of any status related to the VNF. Furthermore, we will study the different use cases of our platform, such as enabling the provision of network access connectivity to users in remote areas, considering the interaction of SUAVs with other NFV enabled ground-units.

## Figures and Tables

**Table 1:** Simulation parameters.

Parameter	Values
Traffic	Constant Bit Rate
Transmission Rate	32 kbps
Network Protocol	UDP
Simulation Time	18,000 s
Number of SUAVs	16
Mobility Model	Static
Failures	Uniform Distribution a=0,b=40
Replacements	Exponential Distribution λ=1/3
Simulation Area	280 m × 280 m
